# Trunk distortion weakens the tree productivity revealed by half-sib progeny determination of *Pinus yunnanensis*

**DOI:** 10.1186/s12870-024-05350-8

**Published:** 2024-07-03

**Authors:** Zhongmu Li, Chengjie Gao, Fengxian Che, Jin Li, Lu Wang, Kai Cui

**Affiliations:** 1 State Key Laboratory of Tree Genetics and Breeding, Institute of Highland Forest Science, Chinese Academy of Forestry, Kunming, 650233 PR China; 2 College of Forestry, Southwest Forestry University, Kunming, 650224 PR China; 3 Tree Breeding Station of Midu County, Midu, 675600 PR China

**Keywords:** *Pinus yunnanensis*, Genetic improvement, Progeny testing forest, Twisted trunk, Family selection

## Abstract

Twisted trunks are not uncommon in trees, but their effects on tree growth are still unclear. Among coniferous tree species, the phenomenon of trunk distortion is more prominent in *Pinus yunnanensis*. To expand the germplasm of genetic resources, we selected families with excellent phenotypic traits to provide material for advanced generation breeding. The progeny test containing 93 superior families (3240 trees) was used as the research material. Phenotypic measurements and estimated genetic parameters (family heritability, realistic gain and genetic gain) were performed at 9, 15, and 18 years of age, respectively. The genetic evaluation yielded the following results (1) The intra-family variance component of plant height (PH) was greater than that of the inter-family, while the inter-family variance components of other traits (diameter at breast height (DBH), crown diameter (CD), height under branches (HUB), degree of stem-straightness (DS)) were greater than that of the intra-family, indicating that there was abundant variation among families and potential for selection. (2) At half rotation period (18 years old), there was a significant correlation among the traits. The proportion of trees with twisted trunks (level 1–3 straightness) reached 48%. The DS significantly affected growth traits, among which PH and DBH were the most affected. The volume loss rate caused by twisted trunk was 18.06-56.75%, implying that trunk distortion could not be completely eliminated after an artificial selection. (3) The influence of tree shape, crown width, and trunk on volume increased, and the early-late correlation between PH, DBH and volume was extremely significant. The range of phenotypic coefficient of variation, genetic variation coefficient and family heritability of growth traits (PH, DBH, and volume) were 44.29-127.13%, 22.88-60.87%, and 0.79–0.83, respectively. (4) A total of 21 superior families were selected by the method of membership function combined with independent selection. Compared with the mid-term selection (18 years old), the accuracy of early selection (9 years old) reached 77.5%. The selected families’ genetic gain and realistic gain range were 5.79-19.82% and 7.12-24.27%, respectively. This study can provide some useful reference for the breeding of coniferous species.

**Supplementary Information:**

The online version contains supplementary material available at 10.1186/s12870-024-05350-8.

## Introduction

*Pinus yunnanensis*, a type of evergreen coniferous tree belonging to the Pinaceae family and *Pinus* genus, is a major timber and afforestation species in southwest China. It is adaptable and can thrive on poor rocky terrain or barren hillsides that other trees cannot grow on, effectively preventing soil erosion [1, 2]. It has a wide range of uses including construction, sleepers, lumber and furniture. It can be used as a raw material for the wood fiber industry. Moreover, it is rich in resin, and its different tissues can be used to process various chemical products, such as tannin extract and oil extraction. In addition, its roots can cultivate rare medicinal herbs [3, 4]. Thus, it plays a vital role in forest ecosystem security and forest product production. However, the genetic degradation of *P. yunnanensis* is a prominent problem with a high proportion of twisted trunks, and corresponding varieties and ecological types were produced, such as *P. yunnanensis* var. *pygmaea*. Its natural regenerative ability is strong, exacerbating the genetic degradation problem [5, 6]. Therefore, it is important to carry out the selection of superior families to prevent the further genetic degradation.

In forest tree breeding programs, accurately estimating the genetic parameters of traits is critical for formulating breeding strategies [7]. Heritability is an important parameter for estimating the degree to which a trait is controlled by genetics, while genetic gain is a crucial indicator of how well a trait is transmitted to offspring [8–10]. Diao et al. (2016) [11] conducted genetic parameter and early selection studies on open-pollinated families of Japanese larch (*Larix kaempferi*) and found that the individual, family heritability and early-late correlation of plant height and diameter at breast height exhibited certain dynamic changes with age, and the optimal early selection ages for plant height and diameter at breast height were 4 and 5 years, respectively. However, due to differences in research materials, tree ages, study site and calculation methods, genetic parameters obtained for the same hybrid type in different studies are often not the same, especially for the genetic performance of growth traits at different ages [12, 13]. Consequently, conducting continuous genetic analyses of data from the same batch of trial materials over multiple years and over multiple sites can provide a more comprehensive understanding of their genetic characteristics.

The selection of superior families is an important method for forest tree breeding and the most basic work for genetic improvement [14–16]. Its results can provide superior germplasm for forest cultivation, and scientific guidance for the breeding of improved varieties, and is of great significance for realizing genetic improvement of forest trees [17, 18]. Zhang et al. (2020) [19] select superior families of *L. olgensis* to establish advanced second-generation seed orchards. Yang at al. (2020) [20] select superior families of *P. massoniana* to serve as material for large-diameter construction timber. An important issue in family selection is selection time, which is also the most widely studied aspect of family selection. Early selection of families can shorten the breeding cycle and accelerate the breeding process [21, 22]. Foster (1987) [23] analyzed that early selection of tree height is effective by the research results of coniferous trees such as loblolly pine. Li et al. (2011) [24] found that the height and diameter of *L. gmelinii* seedlings showed a significant correlation with their future field growth performance during the growth seasons of the first and second years. However, early growth of trees may not fully reflect their later growth performance. Based on the growth dynamics from 1 to 26 year old of *P. Contrata*, Hayatgheibi et al. (2017) [25] disclosed tree traits (diameter at breast height, ring width, and wood density) of different families change with increasing forest age. Yuan et al. (2022) [26] analyzed the dynamic growth performance of superior trees of *Quercus acutissima* sources and found that the provenances with better growth in the early stage showed general performance in the later stage. Premature selection of families or provenances may lead to the misselection or omission of some superior provenances or families [27]. Therefore, studying the changes in tree growth with age and revealing the age effects of growth and material properties can reduce the bias caused by early selection in juvenile periods and provide data support for determining the optimal early selection age.

In summary, this study was based on the phenotypic data of 9a, 15a and 18a in the progeny test of the first generation seed orchard of *P. yunnanensis*, and the research objectives were to (I) assess the relationship between genetic parameters and forest age; (II) To evaluate the effect of early selection; (III) to screen the impact of the trunk distortion after an artificial selection; (IV) Selection of superior families according to phenotypic information and genetic parameters. This is work aim to provide a theoretical basis and excellent germplasm materials for the genetic improvement of *P. yunnanensis*.

## Materials and methods

### Study materials and experimental design

The progeny test forest is located in the seed orchard of *P. yunnanensis* in Midu County, Yunnan Province, China (100°28’E, 25°27’N), at an elevation of 1900–2000 m. The site belongs to the subtropical monsoon climate zone of central Asia, with an average annual temperature of 16.2℃, a maximum temperature of 34.5℃, and an extreme minimum temperature of -6.8℃. The annual rainfall is 752 mm, concentrated mainly from June to October. The soil is mountain red soil, with a pH of 6.0. The experiment began in March 2003 with seedling cultivation and tree planting in July and August 2004, with a spacing of 2 m × 3 m between trees. The progeny test was carried out using seeds from 1 to 93 clones as the test unit. The progeny test forest was designed according to random block. In each block, 6 plants of each family were arranged in rows, and 93 families were randomly arranged in the block. The block was repeated 10 times. That is, a total of 60 plants were planted in each family. There are differences of survival rates among different families (Table S1). The progeny test forest was divided into 3 regions that including 3, 4 and 3 blocks, respectively.

### Data survey

A forest inventory was conducted in 2013, 2019 and 2022, respectively. We measured all the trees in each family, with a total of 3240 trees. The survival rate of each family is shown in Table S1. Six phenotypic traits that were genetically stable [2], easy to obtain and measure were adopted, including plant height (PH), diameter at breast height (DBH), long crown diameter (LCD), short crown diameter (SCD), height under the branches (HUB), and degree of stem-straightness (DS). Only PH and DBH traits were measured in 2013 and 2019, and six traits were measured in 2022. The PH and HUB were measured with a tree altimeter (height measuring device LD6172) (accuracy of 0.01 m). DBH was measured with a vernier caliper (accuracy of 0.01 cm). In addition, the crown diameter was measured with a tower rule (accuracy of 0.01 m), which was the mean value along the slope and contour. LCD and SCD were referred to as the maximum and minimum tree crown diameter, respectively. The DS was assessed based on the level of wood distortion, specifically the deviation angle between the phloem texture and the vertical growth direction of the trunk (Figure S1). The following scoring method was used (level 5: 0°; level 4: 1–5°; level 3: 6–10°; level 2: 11–15°; and level 1: ≥15°). The wood volume was calculated using the binary forest volume Eq. [3], as follows:

1 $$V=a\times {D}^{b}\times {H}^{c}$$

In Eq. (1), where *D* is the diameter at breast height and *H* is the plant height, the coefficients are a = 0.000058290117, b = 1.9796344, and c = 0.90715154. This study measured each tree in the experimental forest three times for each of the selected traits.

### Standardized data

The units and dimensions of 7 phenotypic indicators investigated in this study are inconsistent. To comprehensively compare the differences among different family, the phenotypic indicators were normalized to the range from − 1 to 1. The conversion formula is as follows:

2 $$U=2\times \left(T-{T}_{min}\right)/\left({T}_{max}-{T}_{min}\right)-1$$

*T* represents the measured value of each individual, *T*_*max*_ and *T*_*min*_ represent the measured maximum and minimum values, respectively.

### Data processing

Nested variance analysis was used to study the significance of differences in phenotypic traits among and within families. The linear model used was:

3 $${y}_{ijn}=\mu +{\alpha }_{i}+{\beta }_{j\left(i\right)}+{\epsilon }_{ijn}$$

In Eq. (3), *y*_*ijn*_ represents the *n* observations value of the *j* individual of the *i* family, *µ* means overall average, *α*_*i*_ is the fixed effect value of the *i* family, *β*_*j(i)*_ is the random effective value of the *j* individual in the *i* family, and *ε*_*ijn*_ is the experimental error of the *ijn* observation value.

A mixed linear model was used to analyze the variance components of the growth traits. The following model was used:

4 $${y}_{ijk}=\mu +{B}_{i}+{f}_{j}+{fb}_{ij}+{e}_{ijk}$$

In Eq. (4), ${y}_{ijk}$ is the observation on the k^th^ tree in the j^th^ family in i^th^ block, $\mu$ is the overall mean, *B*_*i*_ is the fixed effect of i^th^ block, *f*_*j*_ is the random effect of j^th^ family, *fb*_*ij*_ is the reciprocal effect of the i^th^ family and j^th^ block, and *e*_*ijk*_ is error.

The equation of loss of phenotypic traits is:

5 $$P={P}_{5}-{P}_{i}$$

The equation of loss rate of phenotypic traits is:

6 $${R}_{P}=P/{P}_{5}$$

In Eq. (5) to (6), *P*_*5*_ represents phenotypic traits of grade 5 straightness, and *P*_*i*_ represents phenotypic traits of straightness at all levels.

The family heritability (narrow-sense heritability) [19]:

7 $${h}_{f}^{2}={\sigma }_{f}^{2}/(\frac{{\sigma }_{e}^{2}}{bn}+\frac{{\sigma }_{fb}^{2}}{b}+{\sigma }_{f}^{2})$$

Genetic variation coefficient [19]:

8 $${V}_{CVG}=\sqrt{{\sigma }_{f}^{2}}/\stackrel{-}{X}\times 100\text{\%}$$

Phenotypic coefficient of variation [19]:

9 $${V}_{CVP}=\sqrt{{\sigma }_{P}^{2}}/\stackrel{-}{X}\times 100\text{\%}$$

In Eq. (7) to (9), b is the number of blocks, n is the number of trees in a block, ${\sigma }_{f}^{2}$ represents the family variance, ${\sigma }_{e}^{2}$ represents the random error, ${\sigma }_{fb}^{2}$ represents the family by block interaction variance and ${\sigma }_{P}^{2}$ represents the phenotypic variance. $\stackrel{-}{X }$represents the mean value of the trait for the population [28].

The calculation formula of phenotypic correlation coefficient [11]:

10 $${r}_{p}={Cov}_{p12}/\sqrt{{\sigma }_{p1}^{2}\times {\sigma }_{p2}^{2}}$$

In Eq. (10): *Cov*_*P12*_ is the phenotypic covariance of traits *X*_*1*_ and *X*_*2*_, ${{\sigma_{P1}}}^{2}$and${{\sigma_{P2}}}^{2}$ is the phenotypic variance of traits *X*_*1*_ and *X*_*2*_.

Genetic gain [11]:

11 $$\varDelta G=\frac{{X}_{i}-\stackrel{-}{X}}{\stackrel{-}{X}}\times {h}_{f}^{2}\times 100\text{\%}$$

Realistic gain [11]:

12 $$G=\frac{{X}_{i}-\stackrel{-}{X}}{\stackrel{-}{X}}\times 100\text{\%}$$

In equations (11) to (12), *X*_*i*_ represents the mean value of the trait for the family.

Membership function:

13 $${\mu }_{A}=({X}_{i}-{X}_{min})/({X}_{max}-{X}_{min})$$

In equations (13), *X*_*min*_ represents the minimum value of the selected trait, while *X*_*max*_ represents the maximum value of the selected trait.

The structural equation model is used to determine the weight of the volume index. It is divided into measurement model and structural model [29].

14 $$X={}_{x}\xi +\delta$$

15 $$Y={}_{y}{\upeta }+e$$

16 $$\eta =B\eta +\xi +\zeta$$

The Eqs. (14) and (15) are the measurement model, *X* is the measurement variable of *ξ*, *Y* is the measurement variable of *η*, *ξ* is the exogenous latent variable, *η* is the endogenous latent variable, *δ* and *ε* are the measurement error vector, *Λ*_*x*_ and *Λ*_*y*_ are the correlation coefficient matrix of the measurement variables *X*, *Y* and the latent variables *ξ* and *η*. Equation (16) is a structural model, which represents the causal relationship between latent variables. *B* is the correlation coefficient matrix between endogenous latent variables, *Γ* is the effect of exogenous latent variable *ξ* on endogenous latent variable η. *ζ* is the part that cannot be explained in the model, which is the error of endogenous latent variables.

The ANOVA, Duncan’s multiple comparisons and PCA were performed using SPSS 21.0. R package (Performance Analytics) was used to correlation analysis [30]. The structural equation model was calculated using AMOS24.0 software. Heatmap analysis was performed using Genesis software.

## Results

### Family variation of growth traits in *P. Yunnanensis*

In order to explore the significance of differences in various traits among and within families of 18 years old progeny test forest, a variance analysis was conducted (Table 1). The results showed that, except DBH, the differences in all other traits were significant among and within families. This suggests that there is abundant variation in the various traits of 18 years old progeny test forest among and within families, which provides space for the selection of superior families. It is feasible to select superior families from progeny trial. The variance component results of various traits showed that the intra-family variance component of PH was greater than the inter-family variance component, while the inter-family variance components were greater than the intra-family variance component for the other traits. This suggests that PH is more influenced by the intra-family effects, while other traits are more influenced by the inter-family effects.

**Table 1:** Descriptive statistics and variance analysis of various traits

Traits	F		Variance component		
	Among family	Within family	Among family	Within family	Random error
PH	4.104**	4.296**	9.62	10.18	80.20
DBH	3.679**	0.893	9.48	2.33	88.19
LCD	4.247**	1.626**	10.59	4.10	85.31
SCD	3.722**	1.627**	9.41	4.16	86.44
HUB	3.158**	3.046**	7.81	7.61	84.58
DS	2.981**	2.967**	7.58	4.51	87.91
V	2.503 × 10^− 7^**	1.158 × 10^− 7^**	10.95	3.33	86.03

**: *P* < 0.01; *: *P* < 0.05. DBH: diameter at breast height; DS: degree of stem-straightness; HUB: height under the branches; LCD: long crown diameter; PH: plant height; SCD: short crown diameter; V: Volume

### Relationship among various growth traits in *P. Yunnanensis*

**Fig. 1:**
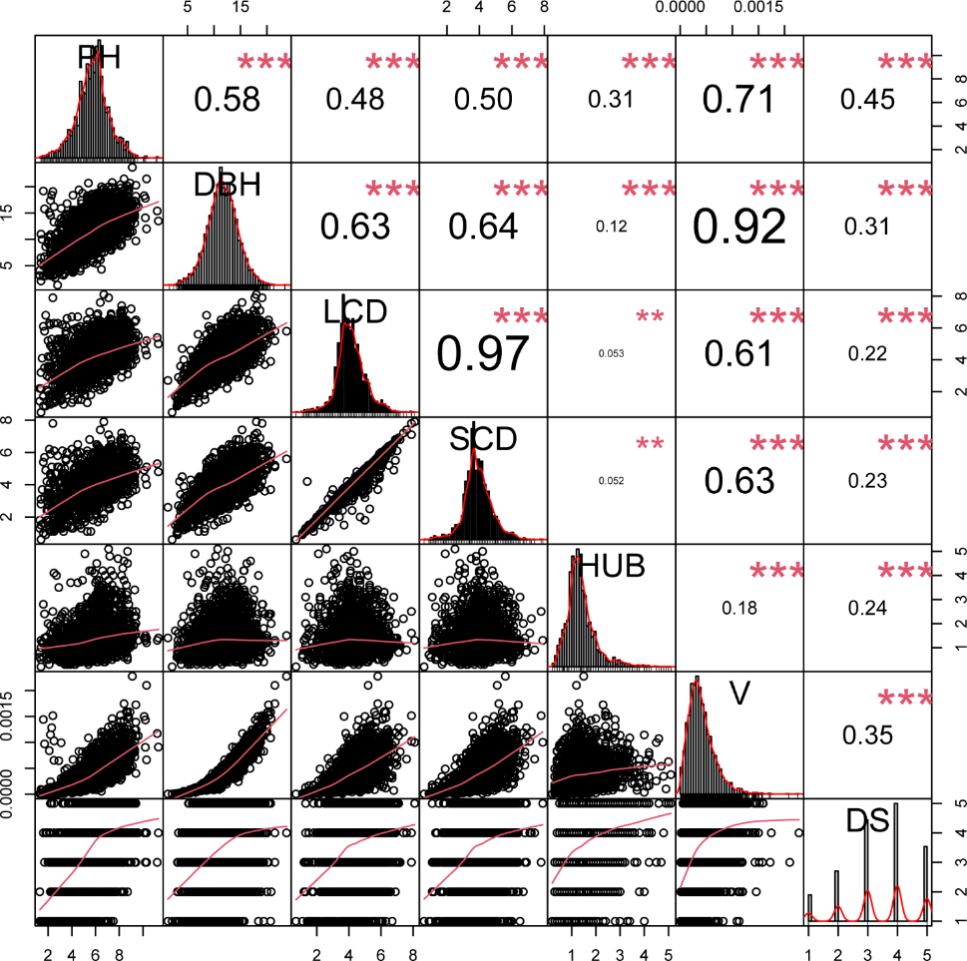
Correlation analysis among phenotypic traits. ***: *P* < 0.001; **: *P* < 0.01; *: *P* < 0.05. DBH: diameter at breast height; DS: degree of stem-straightness; HUB: height under the branches; LCD: long crown diameter; PH: plant height; SCD: short crown diameter; V: Volume

The correlation analysis of 7 traits was carried out (Fig. 1). The PH is highly correlated with DBH and volume, but less correlated with LCD, SCD, HUB and DS. DBH is highly correlated with LCD, SCD and volume, but less correlated with HUB and DS. The HUB is less correlated with DS and volume. These results indicate that the traits such as tree shape, straightness and volume are correlated, and the joint selection of traits can be carried out.

### Factors influencing the volume growth of *P. Yunnanensis*

The reliability and validity test on 7 phenotypic traits showed that the Cronbach’s alpha was 0.849, which was greater than 0.5 and met the requirements of the volume model for further testing. The KMO of the validity test was 0.708, which was greater than 0.7, and the data *P* was 0.000. The Bartlett’s sphericity test had good results, indicating that the data was suitable for factor analysis.

**Fig. 2:**
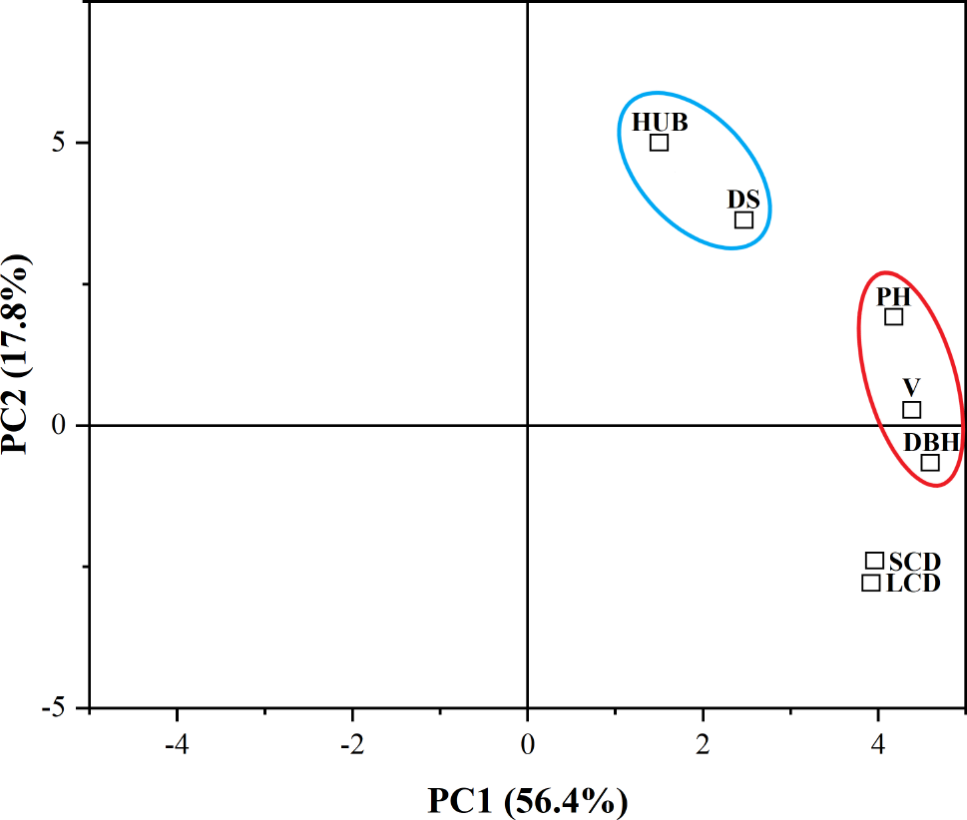
Principal component analysis of phenotypic traits of *P. yunnanensis.* DBH: diameter at breast height; DS: degree of stem-straightness; HUB: height under the branches; LCD: long crown diameter; PH: plant height; SCD: short crown diameter; V: Volume

In order to simplify and classify the 7 traits, principal component analysis (PCA) was conducted (Fig. 2), and two principal components were extracted (eigenvalue > 1), with a cumulative variance contribution rate of 74.2%. The first principal component explained 56.4% of the variance and was mainly dominated by economic traits such as DBH, volume, and PH, while the second principal component explained 17.8% of the variance and was mainly dominated by HUB and DS. The results showed that the first principal component represented 76.01% of the total information of the seven traits. Considering that *P. yunnanensis* is a timber species, economic traits such as PH, DBH, and volume were focused in this study.

**Fig. 3:**
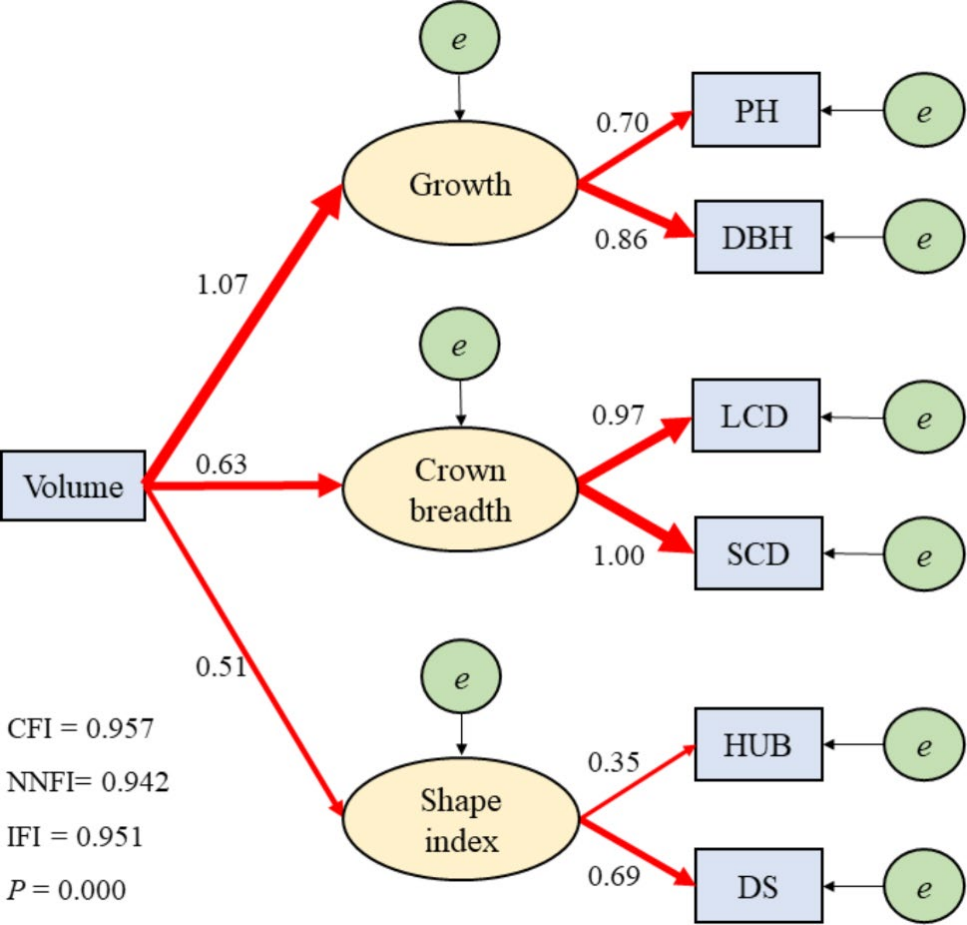
Volume model path. Red arrows indicate significant positive correlation of path relationships, and black arrows indicate errors. Arrow thickness represents the relative size of the standardized path coefficients. DBH: diameter at breast height; DS: degree of stem-straightness; HUB: height under the branches; LCD: long crown diameter; PH: plant height; SCD: short crown diameter

The model was modified according to the M.I. index of the AMOS24.0 output report. The revised model fitness results were shown in Fig. 3, with CFI, NNFI, and IFI being 0.957, 0.942, and 0.951, respectively, all greater than 0.90, and the fitting indexes were in line with the adaptation reference value [31]. The path of the revised structural equation model is shown in Fig. 3. The path coefficients of the endogenous latent variable growth index, crown breadth width index, and shape index were 1.07, 0.63, and 0.51, respectively. The path coefficients of PH and DBH on the growth index were 0.70 and 0.86, respectively. The path coefficients of LCD and SCD on the crown breadth width index are 0.97 and 1.00, respectively. The path coefficients of HUB and DS on the shape index are 0.35 and 0.69, respectively. This indicates that the main factor affecting volume is the growth index, while the crown breadth index and shape index also have an impact on it. Among the growth indices, DBH has a greater impact on growth than PH, which further illustrates that DBH is the main factor affecting volume.

### The impact of trunk twist on the volume of *P. Yunnanensis*

**Fig. 4:**
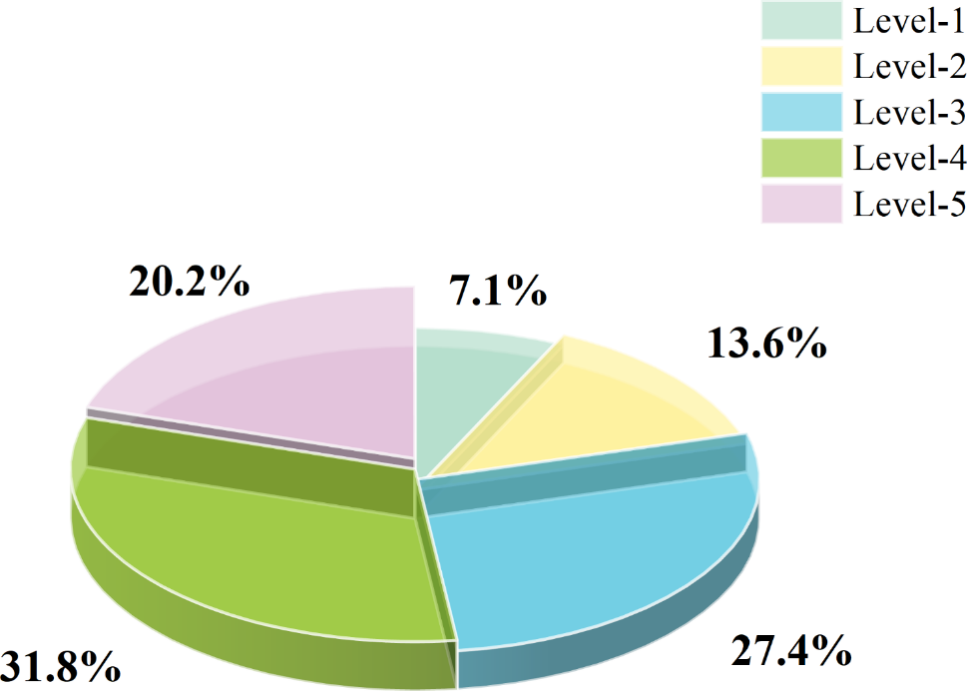
Proportion of *P. yunnanensis* with different degree of stem-straightness. According to the angle between the phloem texture and the vertical growth direction of the trunk, all individuals were divided into 5 levels. Level-5: 0°; Level-4: 1–5°; Level-3: 6–10°; Level-2: 11–15°; Level-1: > 15°

According to the proportion with different DS (Fig. 4), the proportion with straight trunks (levels 4 and 5) (52%) is larger than that with twisted trunks (levels 1, 2 and 3) (48%). Among them, the proportion of level 4 was the highest (31.8%), and the proportion of level 1 was the lowest (7.1%). The object of our survey is the progeny test forest of the first generation seed orchard, which is selected by an artificial selection to eliminate the individual with twisted trunk, implying that trunk distortion could not be completely eliminated after an artificial selection.

**Fig. 5:**
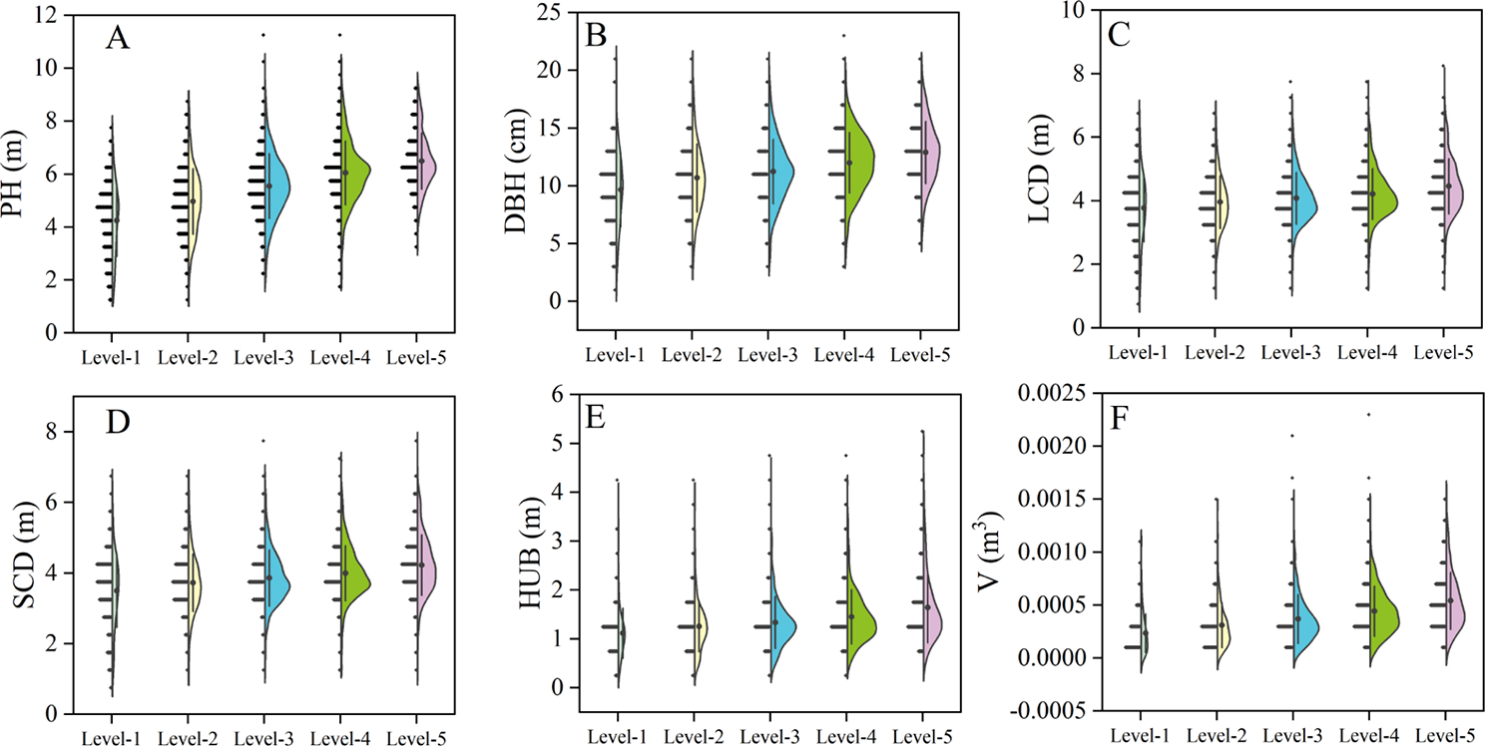
Phenotypic traits of different degree of stem-straightness of *P. yunnanensis.* Level-1 to 5 in the abscissa represent the individual sets of different straightness grades respectively. DBH: diameter at breast height; HUB: height under the branches; LCD: long crown diameter; PH: plant height; SCD: short crown diameter; V: Volume

According to the phenotypic traits with different DS (Fig. 5), PH, DBH, LCD, SCD, HUB and volume showed increasing trend with the increase of DS. In this work, the phenotypic difference between the most straightforward type (level 5) and other levels was defined as loss. The loss rate of PH was 6.84-34.56%, and that of volume reached 18.06-56.75% (Table 2). This indicates that DS positively affects the growth of phenotypic traits, that is, the higher DS, the better the phenotypic traits. Of which, the DS has the greatest influence on PH, DBH and volume.

**Table 2:** Loss of phenotypic traits in *P. yunnanensis* with different degree of stem-straightness

Degree of stem-straightness	PH (m)		DBH (cm)		LCD (m)		SCD (m)		HUB (m)		V (m^3^)	
	Loss	Loss rate	Loss	Loss rate	Loss	Loss rate	Loss	Loss rate	Loss	Loss rate	Loss	Loss rate
Level-1	2.24	34.56%	3.24	33.56%	0.68	18.07%	0.73	17.20%	0.52	31.89%	0.000306	56.75%
Level-2	1.52	23.44%	2.19	17.02%	0.50	13.21%	0.50	11.83%	0.39	23.59%	0.000231	42.81%
Level-3	0.95	14.60%	1.66	12.84%	0.38	9.95%	0.37	8.68%	0.30	18.44%	0.000171	31.72%
Level-4	0.44	6.84%	0.89	6.94%	0.24	6.46%	0.23	5.46%	0.19	11.40%	0.000098	18.06%

The level-5 represents the stem is completely straight, which is the ideal growth state. The data in the table show the gap of the phenotypic indicators between the levels 1–4 and level-5. DBH: diameter at breast height; HUB: height under the branches; LCD: long crown diameter; PH: plant height; SCD: short crown diameter; V: Volume

### Estimation of genetic parameters of main economic traits at different ages

The results of genetic variation analysis for PH, DBH and volume of 93 families at different ages (Table 3) show that the average growth in PH, DBH, and volume increased from 2.94 m, 9.21 cm, and 1.44 × 10^− 4^m^3^ at 9 years old to 5.70 m, 11.55 cm, and 4.03 × 10^− 4^m^3^ at 18 years old, respectively. The genetic variation coefficient and phenotypic coefficient of variation of each trait generally showed a decreasing trend with age (except the genetic variation coefficient of DBH), indicating that each trait gradually tends to be stable with age. The family variance, environmental variance, and variance results of the interaction between family and environment generated by the mixed linear model are presented in Table S2. The ${h}_{f}^{2}$ of each trait did not change significantly with age, indicating that the genetic control level of the main economic traits of *P. yunnanensis* is not affected by age.

**Table 3:** Genetic parameters of economic traits in different forest ages of *P. yunnanensis* families

Trait	Forest age (a)	PH (m)	DBH (cm)	V (m^3^)
Range	18	1.3 ~ 11.3	1.3 ~ 23.7	2.49 × 10^− 6^~2.27 × 10^− 3^
	15	1.2 ~ 11.0	1.0 ~ 22.3	2.20 × 10^− 6^~1.78 × 10^− 3^
	9	0.1 ~ 6.1	1.0 ~ 18.0	3.67 × 10^− 7^~7.95 × 10^− 4^
Mean	18	5.70(1.30)	11.55(2.82)	4.03 × 10^− 4^(2.33 × 10^− 4^)
	15	5.50(1.26)	10.80(2.70)	3.45 × 10^− 4^(2.02 × 10^− 4^)
	9	2.94(0.78)	9.21(2.14)	1.44 × 10^− 4^(8.48 × 10^− 5^)
*V*_*CVG*_ (%)	18	44.29	46.70	123.99
	15	50.72	50.20	121.30
	9	55.19	52.74	127.13
*V*_*CVP*_ (%)	18	22.88	24.30	59.60
	15	23.63	25.15	60.46
	9	26.49	23.22	60.87
${h}_{f}^{2}$	18	0.80(0.00)	0.79(0.01)	0.82(0.02)
	15	0.83(0.02)	0.80(0.03)	0.80(0.05)
	9	0.82(0.00)	0.84(0.00)	0.82(0.01)

*V*_*CVG*_ represents genetic variation coefficient, *V*_*CVP*_ represents phenotypic coefficient of variation, ${h}_{f}^{2}$ represents family heritability. Standard error in brackets

### Early-late correlation of the main economic traits

**Fig. 6:**
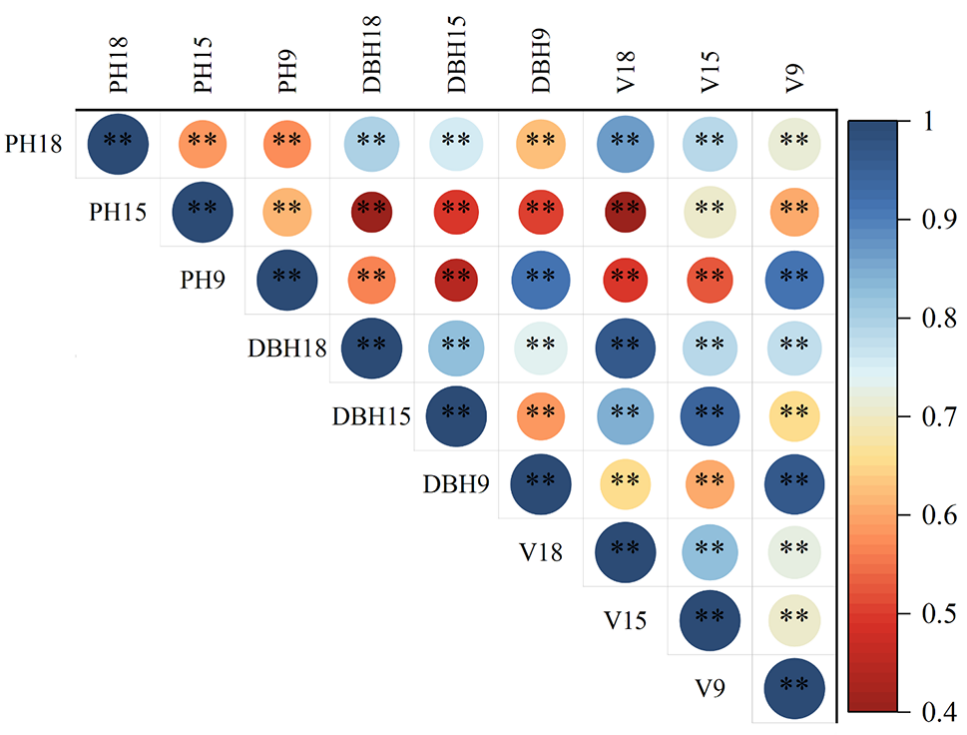
Early and late correlation analysis of economic traits of *P. yunnanensis*. **: *P* < 0.01; *: *P* < 0.05. DBH18: 18 years old diameter at breast height, DBH15: 15 years old diameter at breast height, DBH9: 9 years old diameter at breast height, PH18: 18 years old plant height, PH15: 15 years old plant height, PH9: 9 years old plant height, V18: 18 years old volume of wood, V15: 15 years old volume of wood, V9: 9 years old volume of wood

A correlation analysis was conducted on the PH, DBH, and volume of the 93 families at different ages (Fig. 6). The early and late correlation coefficients of each economic trait showed that the phenotypic correlation coefficients of each trait at the ages of 9, 15, and 18 were significantly positively correlated. The phenotypic correlation coefficients of PH, DBH, and volume between ages were 0.60 ~ 0.64, 0.62 ~ 0.84, and 0.73 ~ 0.84, respectively, indicating that the growth performance of most families was similar between early and late stages, and that conducting early selection of these traits is feasible.

### Superior family selection of *P. Yunnanensis*

**Fig. 7:**
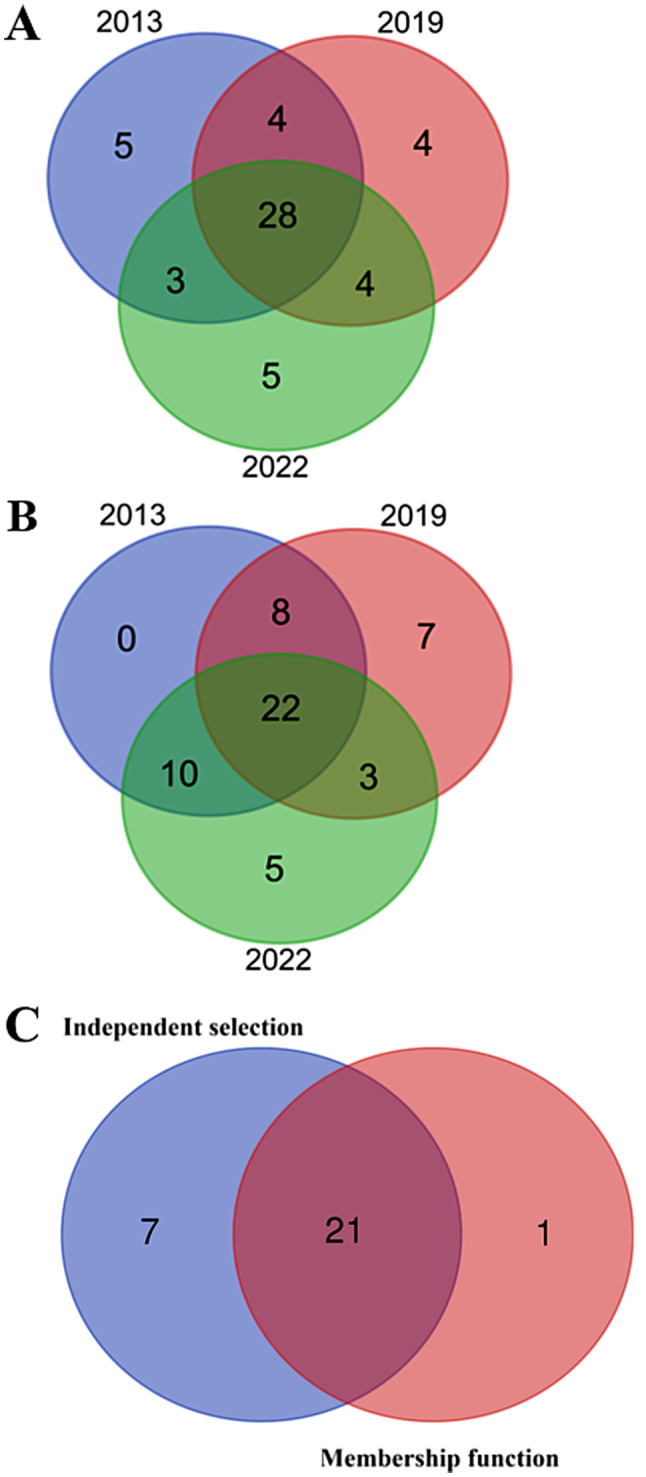
Overlap analysis of superior families of *P. yunnanensis*. The independent selection by volume (**A**), the selection by the membership function (**B**), and the overlap analysis of the membership function and the independent selection (**C**)

**Fig. 8:**
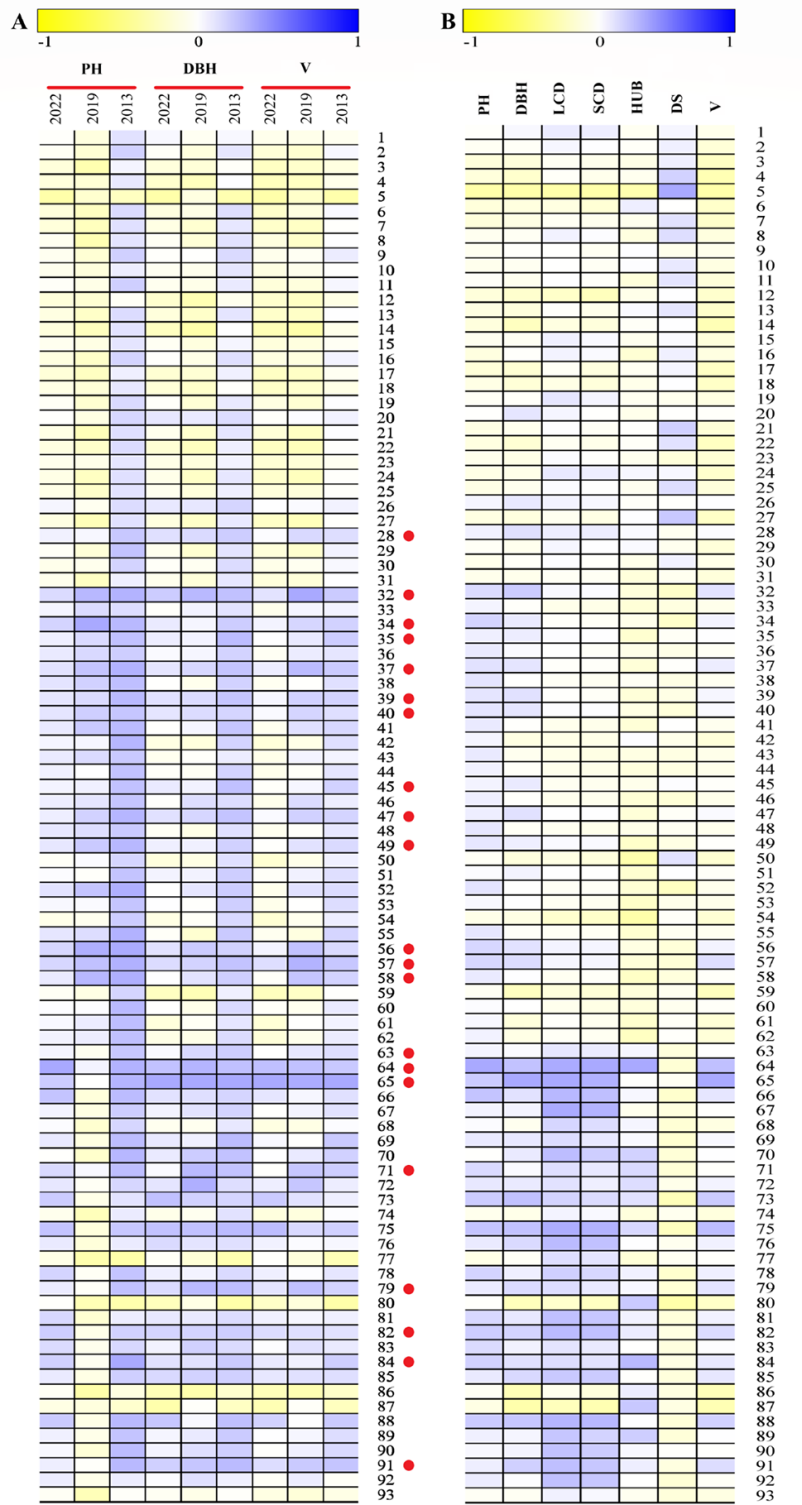
Superior family selection of *P. yunnanensis.* The growth performance of PH, DBH and V of each family at different forest ages. The red circle on the right side of the family number indicates that it is selected as a superior family (**A**). Phenotypic growth indexes of different families in 18 yeasr old (2022) (**B**). Only two indexes of PH and DBH were measured in 2013 and 2019, and six indexes were measured in 2022. All phenotypic indexes were standardized to-1 to 1. In the figure, the closer the blue is, the better the trait is, and the closer the yellow is, the worse the trait is. DBH: diameter at breast height; DS: degree of stem-straightness; HUB: height under the branches; LCD: long crown diameter; PH: plant height; SCD: short crown diameter; V: Volume

As a timber species, volume is the primary concern. The independent selection method was used to select superior families. The families whose volume was ranked in the top 40 above the mean were selected as superior families at the ages of 9, 15 and 18, respectively. The selected superior families of three times were overlapped, and a total of 28 superior families were screened out (Fig. 7A). Meanwhile, membership function method was used to select superior families for the same material, and a total of 22 superior families were screened out (Fig. 7B). Moreover, the superior families selected by independent selection and membership function method was overlapped, and a total of 21 superior families were screened out (Fig. 7C). The growth dynamics of the main economic traits are shown in Fig. 8, and the genetic gain and realistic gain are shown in Table 4. The minimum phenotypic traits of the selected families in the 1/4 rotation period (9 years old) were: PH 2.93 m, DBH 9.43 cm, volume 1.56 × 10^− 4^ m^3^. The minimum phenotypic traits in the 1/2 rotation period (18 years old) were: PH 5.78 m, DBH 11.62 cm, volume 4.28 × 10^− 4^ m^3^. The average genetic gains of PH, DBH, and volume of the selected families increased from 7.65%, 7.67%, and 19.82% in the 1/4 rotation period to 5.73%, 6.50%, and 17.33% in the 1/2 rotation period, respectively. Both genetic and realistic gains decreased with age.

**Table 4:** Genetic gain and realistic gain for economic traits in superior family

Family	PH (%)						DBH (%)						V (%)					
	18		15		9		18		15		9		18		15		9	
	Genetic gain	Realistic gain	Genetic gain	Realistic gain	Genetic gain	Realistic gain	Genetic gain	Realistic gain	Genetic gain	Realistic gain	Genetic gain	Realistic gain	Genetic gain	Realistic gain	Genetic gain	Realistic gain	Genetic gain	Realistic gain
28	1.93	2.40	3.63	4.38	3.60	4.39	6.58	8.37	5.92	7.39	5.45	6.49	9.82	12.05	15.82	19.74	11.45	14.02
32	6.72	8.35	13.68	16.52	9.70	11.84	10.52	13.38	10.91	13.64	9.01	10.73	22.43	27.53	33.49	41.79	23.67	28.98
34	8.20	10.20	15.99	19.31	7.91	9.65	4.47	5.68	1.77	2.21	5.56	6.62	14.16	17.38	16.81	20.97	18.79	23.00
35	2.39	2.97	8.28	10.00	5.69	6.94	3.92	4.99	2.11	2.64	10.62	12.65	8.70	10.68	9.84	12.28	22.25	27.24
37	5.22	6.50	11.73	14.17	9.88	12.06	5.61	7.13	6.29	7.86	8.18	9.73	17.14	21.03	26.86	33.51	23.37	28.61
39	4.55	5.66	8.33	10.06	7.89	9.63	6.34	8.06	5.64	7.04	7.75	9.23	12.88	15.81	18.90	23.59	18.54	22.70
40	4.45	5.54	10.05	12.13	4.28	5.22	5.50	6.99	5.49	6.86	3.12	3.72	10.73	13.17	17.12	21.36	11.20	13.71
45	1.49	1.86	4.74	5.73	5.18	6.31	4.63	5.89	2.25	2.82	9.08	10.81	5.56	6.82	4.88	6.08	19.14	23.43
47	2.59	3.22	9.61	11.61	8.87	10.82	6.37	8.10	6.39	7.99	8.14	9.69	10.89	13.36	18.91	23.59	17.73	21.71
49	4.00	4.98	10.83	13.08	8.15	9.94	3.54	4.50	2.54	3.18	4.34	5.17	6.37	7.81	12.22	15.25	14.02	17.17
56	7.54	9.38	15.05	18.17	12.07	14.73	6.13	7.80	8.16	10.20	4.29	5.10	14.46	17.74	24.33	30.36	14.26	17.46
57	7.66	9.52	12.20	14.73	10.15	12.38	7.33	9.32	6.92	8.64	8.56	10.19	25.41	31.17	30.07	37.51	25.95	31.77
58	3.38	4.20	13.83	16.70	10.79	13.16	0.61	0.78	4.54	5.68	5.29	6.30	4.95	6.08	23.15	28.88	18.37	22.49
63	1.26	1.57	-0.03	-0.03	3.85	4.69	1.43	1.82	5.99	7.49	5.60	6.66	7.43	9.12	11.00	13.72	11.41	13.97
64	17.57	21.85	4.27	5.16	9.97	12.16	12.52	15.92	11.46	14.32	9.27	11.04	40.93	50.22	30.79	24.68	25.01	30.61
65	10.09	12.55	2.33	2.82	9.18	11.20	17.27	21.97	13.19	16.48	15.90	18.93	52.34	64.22	32.46	40.50	44.57	54.57
71	6.84	8.50	3.76	4.54	6.42	7.83	1.31	1.67	10.68	13.34	8.37	9.96	6.05	7.42	22.45	28.01	21.30	26.08
79	3.20	3.97	1.32	1.59	5.61	6.85	7.51	9.55	10.72	13.40	10.03	11.94	22.24	27.28	24.80	30.94	18.23	22.32
82	9.74	12.12	-1.29	-1.55	0.99	1.21	8.95	11.37	6.89	8.61	5.21	6.20	26.39	32.38	13.48	16.82	9.14	11.20
84	8.86	11.02	0.14	0.17	12.38	15.10	6.32	8.04	4.76	5.94	6.40	7.62	19.29	23.67	7.24	9.04	20.73	25.38
91	2.61	3.25	1.67	2.02	8.01	9.77	9.72	12.36	8.92	11.15	10.82	12.88	25.75	31.60	21.42	26.72	27.13	33.21
Mean	5.73	7.12	7.15	8.63	7.65	9.33	6.50	8.27	6.74	8.42	7.67	9.13	17.33	21.26	19.81	24.06	19.82	24.27

DBH: diameter at breast height; DS: degree of stem-straightness; V: Volume

Due to the different growth rates of each family, the selection effect originated from different ages and different selection intensities will be quite different. In this study, 21 superior families were selected based on the combination of membership function and independent selection by volume as the standard to analyze the selection effect at ages 9, 15, and 18. The correct selection rate by the independent selection and membership function methods both decreased with the increase of selection intensity (Table 5). To maintain a correct selection rate over 90%, the selection intensity for early selection of superior families should be mainly focused on the top 35–40 families.

**Table 5:** Effects of independent volume selection method and membership function method in selecting excellent families at different age stages of the experimental forest

Selection criterion		9 years old		15 years old		18 years old	
		Number of selected	Correct selection rate (%)	Number of selected	Correct selection rate (%)	Number of selected	Correct selection rate (%)
Independent selection method	Top 10	8	38.09	9	42.86	7	33.33
	Top 20	15	71.42	16	76.19	11	52.38
	Top 30	20	95.23	19	90.48	16	76.19
	Top 40	21	100	21	100	21	100
Membership function method	Top 10	8	38.10	8	38.10	6	28.57
	Top 20	15	71.43	13	61.90	11	52.38
	Top 30	17	80.95	18	85.71	18	85.71
	Top 40	21	100	21	100	21	100

## Discussion

### Genetic variation of growth traits in *P. yunnanensis* families

The widespread geographical distribution of *P. yunnanensis* results in significant genetic variation in phenotypic traits among different populations [1–3, 32]. Variation of forest traits is the basis for genetic improvement and the prerequisite for family selection, and it determines the potential for improvement in forest [33, 34]. Previous studies showed that tree species with the same genetic structure exhibit significant genetic variation at the individual, family, and provenance levels [35]. In this study, significant differences were found in PH, DBH, LCD, SCD, HUB, DS, and volume among different *P. yunnanensis* families in the progeny test forest. This indicates that there are abundant variations among *P. yunnanensis* families in the progeny test forest, which provided a good basis for the selection of superior families. This result is consistent with the findings of Ji et al. (2007) [15] that there is abundant variation in PH, DBH, volume, and other traits among half-sibling families of *P. massoniana*. The variance component is an indicative indicator that measures the effects on a trait [36]. In this study, the fact that σ_e_ is much greater than σ_af_ and σ_wf_ may be due to environmental factors or other unknown factors. Except PH, other traits showed that σ_af_ > σ_wf_, indicating that the variation in *P. yunnanensis* is mainly affected by family effects, further demonstrating that genetic factors play a major role in the variation among families.

Genetic parameter estimation plays a guiding role in the genetic improvement of forest trees, and heritability is one of the important genetic parameters, which reflects the reliability of selecting genotypes in the population [7, 8, 11]. The selection method and selection intensity can be determined by estimating heritability. Therefore, heritability estimation has important guiding significance for the formulation of breeding strategies [8, 37]. In this study, the ${h}_{f}^{2}$ of the main economic traits of *P. yunnanensis* tended to be stable, ranging from 0.82 to 0.84 at 9 years old to 0.79–0.82 at 18 years old, indicating that the traits of *P. yunnanensis* is under strong genetic control and has stable genetic characteristics. In contrast to this study, the narrow-sense heritability (*h*^2^) of various trait of *L. kaempferi* [11] and *Cunninghamia lanceolata* [38] increased first and then gradually stabilized with age. This difference may be due to differences in the research materials. *P. yunnanensis* is mainly affected by inter-families effects, so its heritability is more stable. The *h*^2^ of DBH in 24 years old *P. elliottii* was 0.27 [39]. and the *h*^2^ of PH in 22 years old *P. sylvestris* was 0.30 [40]. Belaber et al. (2018) [41] reported that the *h*^2^ of DBH and PH in 3 years old *P. elliottii* × *P. caribaea* was 0.34–0.71 and 0.27–0.76, which change to 0.35–0.62 and 0.24–0.55 respectively. Compared with other members of the pine genus, *P. yunnanensis* exhibits higher *h*^2^. This is because the material of this study is the progeny test forest, and its parents are superior trees by artificially selection, displaying excellent in PH, DBH, crown width and other traits. These superior individuals were mated through open-pollination, and some traits have high heritability. In addition, the materials selected in this study are intraspecific hybridization. In the long-term breeding process, if new genetic variation sources are not introduced or proper genetic communication is not carried out, the genetic diversity of the breeding population may be lost, and then the phenomenon of high heritability may occur. Furthermore, some studies shown that the estimated heritability of materials grown under more consistent environmental conditions is higher than that of materials with more different environmental conditions [42].

Genetic variation coefficient is an effective indicator for measuring the genetic variation potential of related growth traits. The size of the coefficient of variation reflects the degree of population dispersion and determines the space for selection [7]. In this study, the phenotypic coefficient of variation ranges for PH, DBH, and volume of *P. yunnanensis* in 9–18 years were 44.29–55.19%, 46.70-52.74%, and 121.30-127.13%, respectively, while the genetic variation coefficient ranges were 22.88–26.49%, 23.22–25.15%, and 59.60-60.87%, respectively. With increase of age, the phenotypic coefficient of variation and genetic variation coefficient showed a decreasing trend. It indicated that with the increase of age, the differences between traits gradually decreased and became more stable. This result is consistent with Diao’s (2016) [11] findings that the phenotypic coefficient of variation and genetic variation coefficient of *L. kaempferi* in PH, DBH, and volume decrease with age. For 8 years old *P. massoniana*, the phenotypic coefficient of variation for PH, DBH, and volume were 11.77%, 22.15%, and 45.27%, respectively, while the genetic variation coefficient were 4.57%, 6.63%, and 13.11%, respectively [15]. The phenotypic coefficient of variation for PH and DBH of 2–30 years old *L. kaempferi* were 14.89–35.65% and 19.17–23.86%, respectively [43]. The phenotypic coefficient of variation for PH and DBH of 12 years old *P. radiata* were 3.8–14.3% and 6.5–25.5%, respectively [44]. Compared with other members of the pine genus, *P. yunnanensis* has higher coefficients of variation. This may be due to the influence of external environmental conditions or other factors (artificial harvesting, grazing and fertilizing, etc.), which makes the families selected in this study undergo stable and heritable variation in the long-term genetic evolution process, showing large phenotypic differentiation and gene differentiation, and finally large phenotypic variation coefficient and genetic variation coefficient. These indicating that it has greater potential for genetic improvement through selection.

### Correlation and early selection of growth traits of *P. Yunnanensis*

The correlation between forest tree traits reflects the relationship between various traits. By analyzing the correlation between traits, we can understand the degree of correlation between traits and weigh the traits in selection, which is beneficial to genetic improvement of forest trees and improving selection efficiency [45]. The results of this study found that there is a highly significant positive correlation between each growth trait of *P. yunnanensis*. The correlation coefficient between PH, DBH, and volume is relatively high, and the correlation coefficient between PH, DBH, and volume and DS is also higher than that of other traits, indicating that the straightness of the trunk positively affects the productivity of trees. On the contrary, twisting trunk will weaken the productivity of trees. In this study, the loss rate of each trait caused by trunk distortion reached 18.06-56.75%. Twisted or deformed tree trunk will reduce their economic value and increase the cost of wood operation and transportation. Therefore, while improving the PH, DBH, and volume of *P. yunnanensis*, the DS has also been indirectly improved. This result is consistent with the findings of *P. massoniana* [15] and *P. radiate* [46], which found that PH, DBH, and volume were highly positively correlated with DS. However, Belaber et al. (2018) [41] found that there was no correlation between PH and DBH and DS in *P. elliottii* × *P. caribaea*. The differences in the above results may be due to differences in the research materials. The heterosis produced by hybridization will change the relationship between phenotypic traits of plants. Blada (1992) [47] crossed *P. strobus × P. griffithii*, the diameter, basal area and volume growth of the hybrids were higher than *P. griffithii*, which changed the relationship between traits. The mechanism of the relationship between DS and PH, DBH, and volume of forest trees needs further study.

Breeding cycles for forest trees are relatively long due to the control of self-factor and environmental factors, which largely limits the process of genetic improvement of forest trees. Early selection plays an important role in shortening the breeding cycle and accelerating advanced generation breeding [21, 22]. The early-late correlation of growth traits is one of the methods of early selection. Xiang et al. (2003) [48] found that the early selection ages of PH and DBH of *P. taeda* was 3 and 4 years, respectively, and DBH had a higher early-late correlation than PH. Lai et al. (2014) [49] believed that the early selection ages of PH and DBH for *L. kaempferi* were 2 and 5 years, respectively, and the genetic correlation between DBH and volume was higher than that between PH and volume at different ages. In this study, the early-late correlation of PH, DBH, and volume were found to be a significantly positive correlation, indicating that 9 years could be a suitable age for early selection of *P. yunnanensis* families. However, the effective age for early selection for different tree species, families, forest stands or traits may be diverse. For example, Dong et al. (2018) [50] found that 6 years could be used as the age for early selection of DBH of *L. principis-rupprechtii*, and 8–9 years could be used as the age for early selection of PH. Weng et al. (2007) [51] believed that 5–7 years could be used as the age for early selection of volume of *P. banksiana*. Lai et al. (2023) [52] found that the best early selection age of tree-ring width of *P. elliottii* was 9–12 years and the best early selection age of wood density was 7–8 years. Due to different early and late growth characteristics of different families, some families may grow slowly in the early stage and grow fast in the later stage, resulting in differences in the selection of superior families at different age stages. Therefore, premature family selection may result in misselection or omission of some superior families. As the correct selection rate decreases with increasing selection intensity, if early selection is to be carried out, the selection intensity must be reduced and the number of selected families increased. Our study showed that in order to control the correct selection rate of superior families at 90%, and the optimal selection intensity for early selection of *P. yunnanensis* families should be the top 35–40 selected by membership function and independent selection of volume.

### Superior family selection of *P. Yunnanensis*

The selection of superior families requires considering multiple factors, and evaluating based on a single factor can be limiting [53]. The joint selection of multiple traits is an important way to achieve simultaneous improvement of multiple traits in tree, and the membership function method can comprehensively evaluate different traits based on membership values [54, 55]. The independent selection method sets a minimum standard value for each trait and selects families that simultaneously exceed the standard value, which is an ideal method for multi-trait selection [56, 57]. This study used a combination of membership function and independent selection methods to select superior families, selecting 21 superior families from 93 families. The selected families had high genetic and realistic gains, with a mean genetic gain of 5.73%, 6.50%, and 17.33% for PH, DBH, and volume at 18 years, respectively, and a mean realistic gain of 7.12%, 8.27%, and 21.26%. Compared to *P. massoniana* with a genetic gain of 6.10% for PH and 19.23% for volume at 8 years [15] and *L. kaempferi* with a genetic gain of 10.91% for PH and 15.04% for DBH at 17 years [43], the selected families in this study had slightly lower genetic gains. The selected family can try to promote the application in production.

Wood properties are also an important factor in tree evaluation. In this study, only the growth traits were measured and analyzed. In the next step, the wood properties traits will be analyzed continuously. Combined with growth traits and wood properties, evaluation and selection of *P. yunnanensis* families can be carried out in the hope of breeding families that grow rapidly and have superior wood properties. Plant phenotypic traits are the result of the interaction of genotype and environment, and also the comprehensive embodiment of genetic diversity and environmental diversity [58, 59]. Plants of the same genotype often form different phenotypic traits under different environmental conditions [60]. There was a significant interaction between family growth and site, and the relative performance of the same family was different in different sites. In the future, we will carry out multi-site determination to exclude the interaction between genotype and environment, so as to select superior materials with different adaptability.

## Conclusion

In this study, it was showed that trunk distortion of *P. yunnanensis* severely weakened the vegetative growth traits, especially in PH and DBH, leading to a loss rate of 18.06-56.75% in volume. After a generation of artificial selection, the harm of trunk distortion still exists. There was abundant variation among families and potential for selection, of which, PH is more influenced by the intra-family effects, while other traits are more influenced by the inter-family effects. A total of 21 superior families were screened by a method of membership function combined with independent selection. Compared with mid-term selection, early selection proved feasibility and accuracy. These findings provide valuable insights for the breeding of coniferous species, and could contribute to the expansion of germplasm of genetic resources.

### Electronic supplementary material

Below is the link to the electronic supplementary material.

Supplementary Material 1

Supplementary Material 2

Supplementary Material 3

## Data Availability

The datasets used and/or analyzed during the current study are available from the corresponding author on reasonable request.
