# Supplementary material for: Trunk distortion weakens the tree productivity revealed by half-sib progeny determination of Pinus yunnanensis
Source: BMC Plant Biol. 2024 Jul 3;24:629. doi: 10.1186/s12870-024-05350-8 (PMC11221199; doi:10.1186/s12870-024-05350-8)
Supplement: Supplementary file 1 — Supplementary Material 1 [file 12870_2024_5350_MOESM1_ESM.docx]

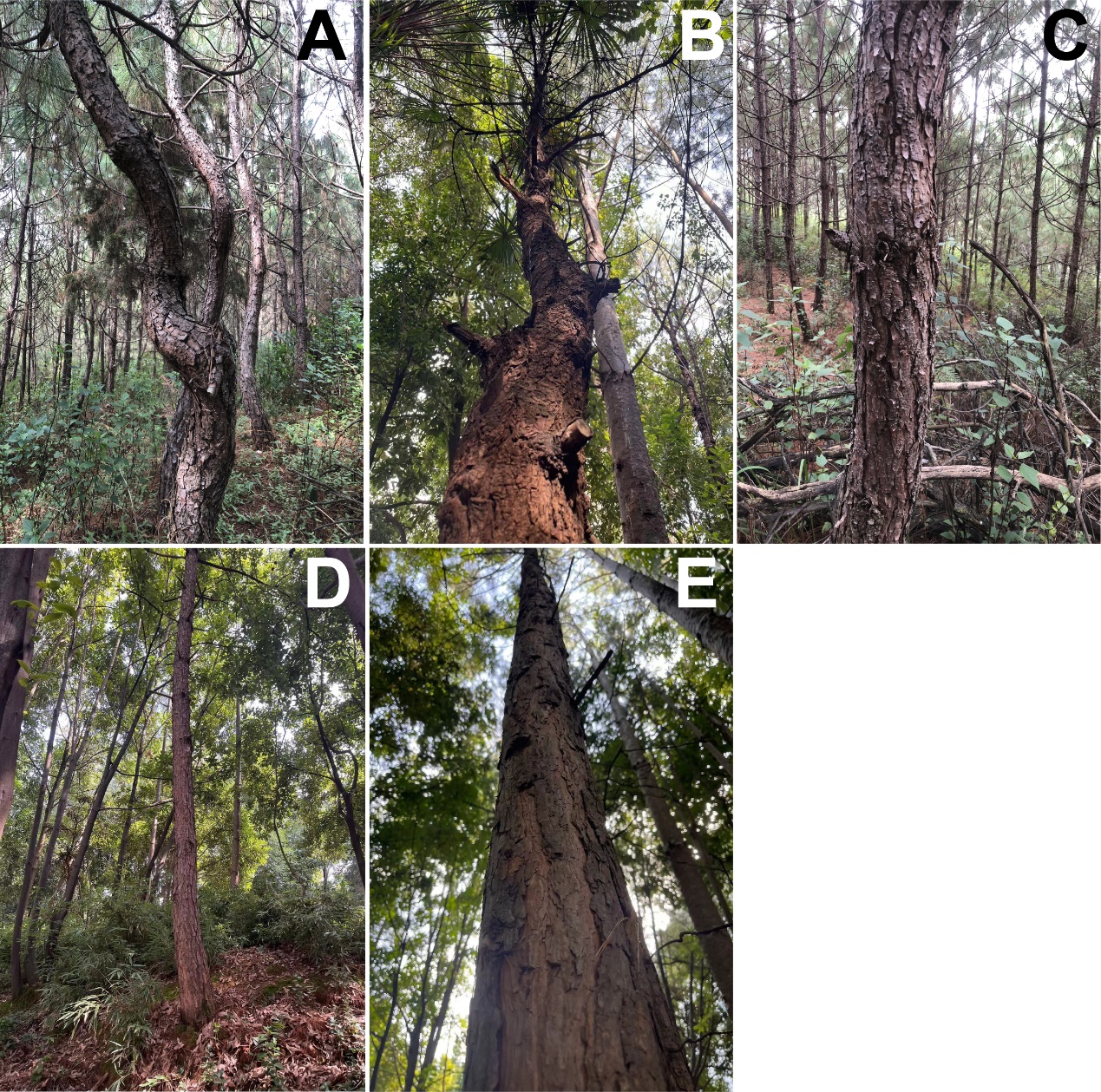


Figure S1. *P. yunnanensis* with different degree of stem-straightness. Level 1 (A), level 2 (B), level 3 (C), level 4 (D), level 5 (E).
