# Supplementary material for: Trunk distortion weakens the tree productivity revealed by half-sib progeny determination of Pinus yunnanensis
Source: BMC Plant Biol. 2024 Jul 3;24:629. doi: 10.1186/s12870-024-05350-8 (PMC11221199; doi:10.1186/s12870-024-05350-8)
Supplement: Supplementary file 3 — Supplementary Material 3 [file 12870_2024_5350_MOESM3_ESM.docx]

Table S2 The variance components of growth traits at different age

| Trait | Forest age (a) | $\sigma_{f}^{2}$ | $\sigma_{fb}^{2}$ | $\sigma_{e}^{2}$ |
| --- | --- | --- | --- | --- |
| PH | 9 | 0.2595 | 0.0754 | 0.9234 |
|  | 15 | 0.3930 | 0.0740 | 1.6479 |
|  | 18 | 0.9046 | 0.4348 | 2.5589 |
| DBH | 9 | 2.6911 | 1.0596 | 4.7181 |
|  | 15 | 3.4397 | 1.4076 | 10.6926 |
|  | 18 | 4.2686 | 2.2544 | 11.2066 |
| V | 9 | 2.069×10^-8^ | 1.821×10^-9^ | 1.147×10^-7^ |
|  | 15 | 8.545×10^-8^ | 4.886×10^-8^ | 1.519×10^-7^ |
|  | 18 | 1.601×10^-6^ | 3.083×10^-7^ | 7.250×10^-6^ |

PH, plant height; DBH, diameter at breast height; V, Volume; $\sigma_{f}^{2}$, $\sigma_{fb}^{2}$and $\sigma_{e}^{2}$represent thevariance components of family, family by block interaction and random error respectively.
